# Cordycepin (3′-deoxyadenosine) suppressed HMGA2, Twist1 and ZEB1-dependent melanoma invasion and metastasis by targeting miR-33b

**DOI:** 10.18632/oncotarget.3383

**Published:** 2015-03-13

**Authors:** Pu Zhang, Changjin Huang, Changliang Fu, Yang Tian, Yijuan Hu, Bochu Wang, Amy Strasner, Yang Song, Erqun Song

**Affiliations:** ^1^ Key Laboratory of Luminescence and Real-Time Analytical Chemistry (Southwest University), Ministry of Education, College of Pharmaceutical Sciences, Southwest University, Chongqing 400715, China; ^2^ Institute of Pathology, Third Military Medical University, Chongqing 400038, China; ^3^ College of Bioengineering, Chongqing University, Chongqing 400030, China; ^4^ Department of Bioengineering, Pennsylvania State University, University Park, PA 16801, USA; ^5^ Division of Hematology/Oncology, Department of Medicine, Robert H. Lurie Comprehensive Cancer Center, Northwestern University Feinberg School of Medicine, Chicago, IL 60611, USA; ^6^ Department of Pathology, Memorial Sloan-Kettering Cancer Center, New York, NY 10021, USA; ^7^ Skaggs School of Pharmacy and Pharmaceutical Sciences, University of California at San Diego, La Jolla, CA 92093, USA; ^8^ Rollins School of Public Health, Emory University, Atlanta, GA 30322, USA

**Keywords:** cordycepin, miR-33b, metastasis, cell migration, focal adhesion

## Abstract

Malignant melanoma, the most deadly form of skin cancer, has a high propensity for metastatic spread and is notoriously chemotherapy-resistant. Cordycepin, the active component of Cordyceps spp., has been identified to have anti-metastatic effect on tumor progression and thus possesses pharmacological and therapeutic potentials. However, the mechanisms of anti-metastatic effects of cordycepin at cellular levels remain elusive. We analyzed the effect of cordycepin on human melanoma miRNA expression profiles by miRNAarray and found that miR-33b was upregulated in highly-metastatic melanoma cell lines following cordycepin exposure. Cordycepin-mediated miR-33b expression was dependent on LXR-RXR heterodimer activation. miR-33b directly binds to HMGA2, Twist1 and ZEB1 3′-UTR to suppress their expression. The negative correlations between miR-33b levels and HMGA2, Twist1 or ZEB1 expression were detected in 72 patient melanoma tissue samples. By targeting HMGA2 and Twist1, miR-33b attenuated melanoma migration and invasiveness upon cordycepin exposure. miR-33b knockdown or ZEB1 overexpression reverted cordycepin-mediated mesenchymal-epithelial transition (MET), triggering the expression of N-cadherin. In spontaneous metastasis models, cordycepin suppressed tumor metastasis without altering primary tumor growth. We showed for the first time that targeting miRNA by cordycepin indicates a new mechanism of cordycepin-induced suppression of tumor metastasis and miR-33b/HMGA2/Twist1/ZEB1 axis plays critical roles in regulating melanoma dissemination.

## INTRODUCTION

Melanoma is the most deadly and malignant cancer insensitive to chemotherapy [1]. Most deaths for melanoma occur from hematogenous dissemination of therapy-resistant tumor disrupting endothelial junction, invading basement membrane and interfering with normal distant organ functions [2–4]. Efforts are, therefore, directed towards development of new anti-metastasis medications.

Cordycepin (or 3′-deoxyadenosine), the bioactive compound present in species of the genus Cordyceps, exhibits a large variety of anti-tumor properties, including cell proliferation inhibition, apoptosis induction, and platelet aggregation inhibition [5, 6] (Supplementary Figure 1A). Cordycepin has also been shown to regulate protein synthesis and cell adhesion by inhibiting kinase activation [7]. In addition, in murine lung injury model, cordycepin suppressed LPS-mediated TNF-α and IL-8 release and cell adhesion molecule expression. While cordycepin exerted no toxicity on normal cell lines, it markedly induced BRCA1-deficient breast cancer death by functioning as a Poly (ADP-ribose) polymerase (PARP) inhibitor [8]. Anti-metastatic activities of cordycepin were demonstrated in mouse models where intraperitoneally administrated cordycepin inhibited B16 mouse melanoma liver metastasis [9]. However, the potential roles of cordycepin in melanoma cell metastasis and the underlying molecular mechanisms are not fully addressed.

microRNAs (miRNAs) are single-stranded non-coding RNAs of 21 to 23 nucleotides, mediating post-translational gene regulation [10, 11]. miRNA targeting is primarily achieved through base-pair interactions between 5′ ends of miRNA and target regions within 3′ untranslated regions (3′UTR) of mRNA [12]. This targeting represses translation or induces cleavage of mRNA. miRNAs have been found to regulate genes involved in diverse biological functions, including development, differentiation, and apoptosis [13, 14]. Cumulating evidence suggested that miRNA plays significant roles in initiation and progression of cancers [15]. The changes of miRNA expression have impact on tumor growth and metastasis by modulating the functions of relevant genes and proteins [16]. Limited information is available regarding the miRNA expression profiles in metastatic melanoma. Overexpression of miR-182 in melanoma cells was associated with increased survival and invasive properties of melanoma cells via regulation of microphthalmia-associated transcription factor (MITF) and O subclass of the forkhead family3 (FOXO3) [17]. miR-221 and miR-222 were also shown to regulate melanoma proliferation and invasion *in vitro* and melanoma growth *in vivo* [18]. miR-214 was found to be overexpressed in metastatic melanoma [19]. By overexpressing miR-214 in low-metastatic cell lines or silencing its expression in highly metastatic cells, it was revealed that miR-214 inhibited melanoma migration, invasion and extravasation *in vitro* and experimental lung metastasis *in vivo*. However, none of these studies attempted to identify the expression profiles of miRNAs and their roles in melanoma metastasis as regulated by natural products.

In the current study, we intended to unveil whether cordycepin may regulate melanoma metastasis via modulating the expressions of miRNAs. We identified a spectrum of miRNAs whose expression profiles were changed by cordycepin treatment. We also found that melanoma migration and invasiveness was mediated by miR-33b though directly targeting high mobility group AT-hook 2 (HMGA2), Twist1 and human zinc finger E-box binding homeobox 1 (ZEB1). Silencing miR33b reverted cordycepin-mediated suppression of invasive, migratory and epithelial-mesenchymal transition (EMT) phenotype *in vitro* and melanoma metastasis *in vivo*. Our study provides a better understanding of the novel action mechanism of cordycepin underlying melanoma metastasis, indicating that miR-33b could be potential targets for melanoma therapy.

## RESULTS

### Cordycepin induces miR-33b expression in melanoma cell lines

**Figure 1:**
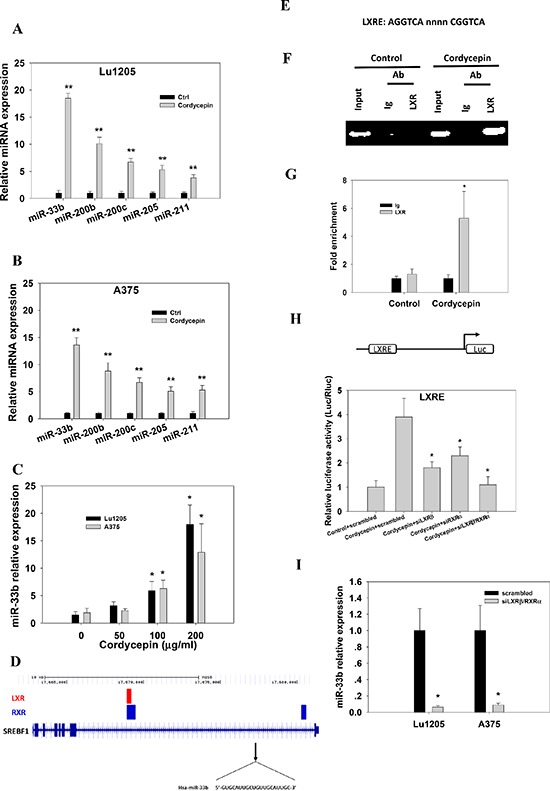
Cordycepin mediates miR-33b expression in melanoma cells in a LXR/RXR dependent manner **(A–B)** qRT-PCR reveals upregulation of miR-33b, miR-200b, miR-200c, miR-205 and miR-211 in Lu1205 and A375 melanoma cells which received 200 μg/ml cordycepin compared with their matched untreated controls. **(C)** Relative expression of miR-33b in Lu1205 and A375 melanoma cells which received 0, 50, 100 and 200 μg/ml cordycepin treatment. Data are expressed as mean±SEM from three independent experiments. *p < 0.05, **p < 0.01 compared with control. **(D)** LXR and RXR binding sites overlap and cluster around target genes. All target genes are shown in their native chromosomal location according to Human Genome Assembly (hg18) in UCSC Genome Browser. Vertical lines in target genomes represent exons, and horizontal lines represent introns. **(E)** Sequences of the consensus LXRE. **(F–G)** 200 μg/ml cordycepin-mediated binding of LXR/RXR to LXRE as measured by chromatin immunoprecipitation assays. The chromatin was extracted from Lu1205 cells treated with or without cordycepin. Crosslinked lysates were immunoprecipitated with control Ig or anti-LXR antibody or not (input). DNA precipitates were isolated and subjected to semiquantitative PCR (F) or quantitative PCR (G) using specific primers covering LXRE. Quantitative PCR data are represented normalized and expressed relative to the levels detected in Ig-immunoprecipitated lysates. Data represent mean ± SEM of 3 independent assays. **(H)** Lu1205 cells were transfected with LXRE luciferase reporter constructs. Cells were treated or untreated with 200 μg/ml cordycepin and transfected with scrambled, siLXRβ, siRXRα and siLXRβ/RXRα before the luciferase activities were measured. Results are relative to the levels in untreated cells. Data represent mean±SEM of 3 independent assays. *p < 0.05 compared with control. **(I)** Relative expression of miR-33b in Lu1205 and A375 melanoma cells which received scrambled or siLXRβ/RXRα followed by 200 μg/ml cordycepin treatment. Data are expressed as mean±SEM from three independent experiments. *p < 0.05 compared with control.

Recent evidence suggested that microRNA plays significant roles in regulating melanoma metastasis [20]. To identify the possible microRNA being changed upon cordycepin treatment, we compared 280 miRNA expression profiles which have been reported to regulate cancer metastasis in two metastatic cell lines, A375 and Lu1205 cells, which were untreated or treated with 100 μg/ml and 200 μg/ml cordycepin. We identified 38 miRNAs whose expression levels were altered by >2-fold following cordycepin treatment. Of the 38 miRNAs, 23 miRNA levels were elevated and 15 miRNA levels were downregulated in both cell lines (Supplementary Figure 1B). Among those, miR-33b, miR-200b, miR-200c, miR-205 and miR-211 exhibited more than 5-fold increase in their expression in response to 200 μg/ml cordycepin. The elevated expressions of miR-33b, miR-200b, miR-200c, miR-205 and miR-211 were verified by realtime-PCR (Figure 1A–1B). Compared with the untreated counterparts, miR-33b had a greater than 18.5-fold higher expression in cordycepin-treated invasive cell lines. Cordycepin boosted miR-33b levels in a dose-dependent manner in both invasive cell lines (Figure 1C). Since miR-33b displayed the most prominent increase, it may be of functional importance in regulating melanoma invasion and metastasis. miR-33b is a member of human miR-33 family and has been reported to regulate lipid metabolism and cholesterol homeostasis by targeting the downstream genes. However, its roles in tumor progression remain elusive. miR-33b is located in intron of SREBP-1 and is expressed when liver X receptor (LXR) binds to LXRE region of SREBP-1 promoter as a heterodimer with the retinoid X receptor (RXR) [21–23] (Figure 1D). To provide a direct evidence that cordycepin induced binding of the LXR protein to LXRE, we employed a chromatin immunoprecipitation assay. In cordycepin-treated cells, LXRE could be amplified by PCR after immunoprecipitation of the fragmented chromatin with an anti-LXR antibody (Figure 1E–1G). To test whether LXR/RXR heterodimers activate the promoter of SREBP-1, LXRE region was cloned into luciferase reporter vector. The promoter activities were increase in Lu1205 cells treated with cordycepin. LXRβ or RXRα silencing partially reduced promoter activities, while simultaneous knockdown of LXRβ and RXRα completely abolished cordycepin-induced luciferase activities (Figure 1H). In addition, silencing LXRβ and RXRα attenuated the induction of miR-33b expression by cordycepin (Figure 1I). These results suggest that cordycepin upregulates miR-33b expression in a LXR/RXR activation-dependent manner.

### miR-33b binds to the 3′ UTR of HMGA2, ZEB1 and Twist1 to regulate their expression in cordycepin-treated melanoma cell lines

**Figure 2:**
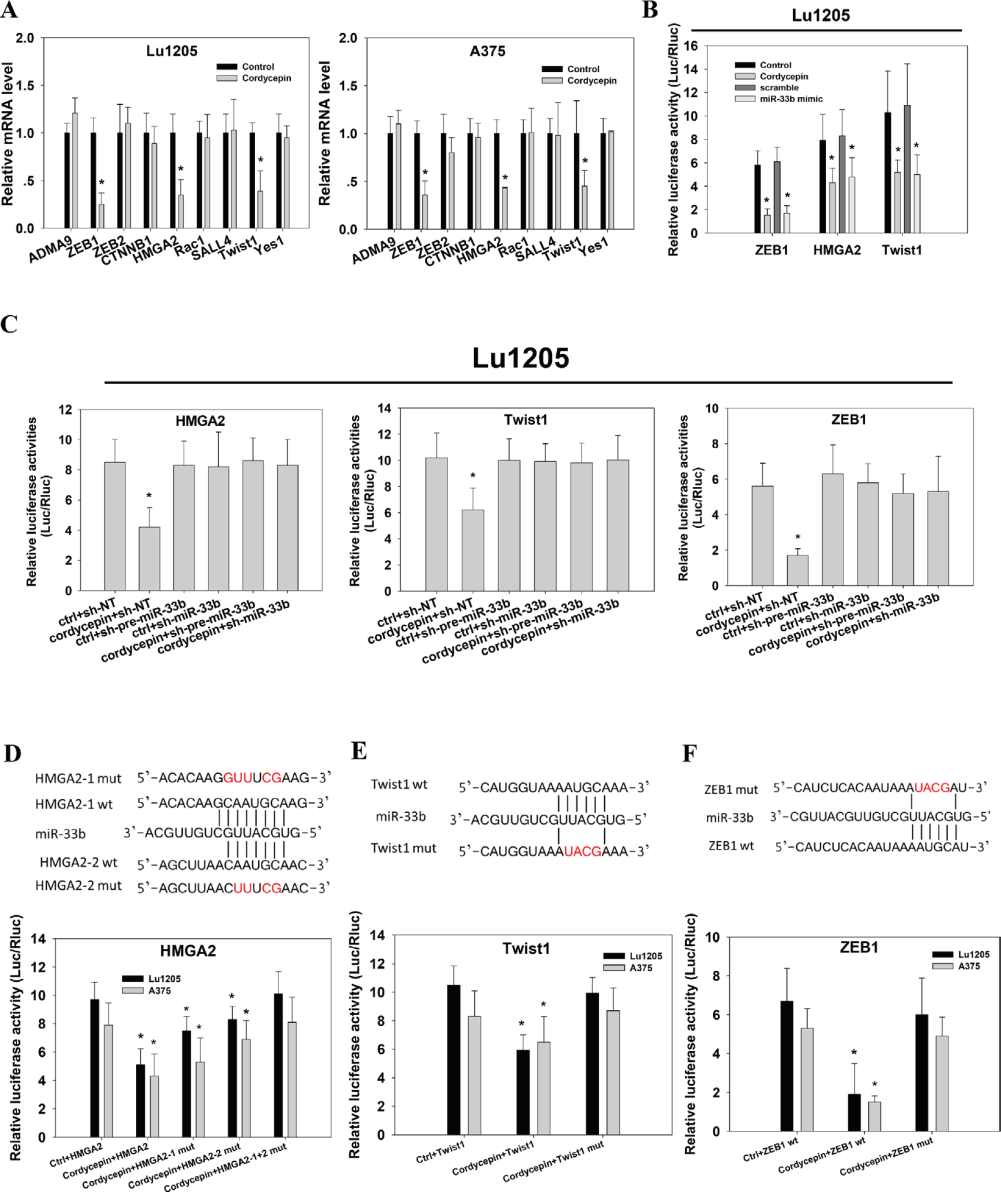
Identification of HMGA2, Twist1 and ZEB1 as the direct targets of miR-33b in response to cordycepin treatment **(A)** The effect of 200 μg/ml cordycepin treatment on ADMA9, ZEB1, ZEB2, CTNNB1, HMGA2, Rac1, SALL4, Twist1 and Yes1 mRNA expression levels in Lu1205 and A375 were measured by qRT-PCR. mRNA levels were normalized against GAPDH reference genes. **(B)** Cordycepin treatment or transient overexpression of miR-33b mimic in Lu1205 reduced luciferase activities of ZEB1, HMGA2 and Twist1 3′UTR reporter. **(C)** miR-33b inhibition reverted cordycepin-mediated suppression of luciferase activities of HMGA2, Twist1 and ZEB1 3′UTR reporter in Lu1205 cells. Lu1205 were transfected with sh-NT, sh-pre-miR-33b or sh-miR-33b for 24 hr before being treated or untreated with 200 μg/ml cordycepin for 24 hr. The relative luciferase activities were measured. **(D–F)** Mutation of miR-33b target sites in HMGA2, Twist1 and ZEB1 3′UTR constructs completely rescued the down-regulatory effect of corycepin on luciferase activity. (D) *Top*, HMGA2 3′-UTR-wt (HMGA2-1 and HMGA2-2 wt) and two 3′-UTR mutants (HMGA2-1 mut and HMGA2-2 mut) in which the sequence in red was mutagenized to abolish miR-33b binding. *Bottom*, luciferase assays showing decreased activity after co-transfection of HMGA2-wt with miR-33b in Lu1205 and A375 cells. The HMGA2-1 mut and HMGA2-2 mut were partially refractory to cordycepin. (E) *Top*, Twist1 3′-UTR-wt (Twist1 wt) and 3′-UTR mutant (Twist1 mut) in which the sequence in red was mutagenized to abolish miR-33b binding. *Bottom*, luciferase assays showing decreased activity after co-transfection of Twist1-wt with miR-33b in Lu1205 and A375 cells. The Twist1 mut were refractory to cordycepin. (F) *Top*, ZEB1 3′-UTR-wt (ZEB1 wt) and 3′-UTR mutant (ZEB1 mut) in which the sequence in red was mutagenized to abolish miR-33b binding. *Bottom*, luciferase assays showing decreased activity after co-transfection of ZEB1-wt with miR-33b in Lu1205 and A375 cells. The ZEB1 mut were refractory to cordycepin. Luciferase activity was assayed and renilla luciferase was used as internal control. Each data are expressed as mean±SEM from three independent experiments. *p < 0.05 compared with control.

To determine the functional roles of cordycepin-upregulated miR-33b in melanoma, we attempted to identify the downstream targets of miR-33b. To narrow down the target genes of miR-33b, we employed *in silico* algorithms to predict miR-33b target genes with binding likehood. Among genes predicted by three major online miRNA target prediction algorithms, namely TargetScan, PicTar and miRanda, we were particularly interested in disintegrin and metalloproteinase domain-containing protein 9 (ADAM9), HMGA2, RAC1, sal-like 4 (SA LL4), Twist1, CTNNB1, ZEB1, ZEB2, Yes1, which had been reported to regulate tumor EMT, stemness, migration, invasion and tumorigenesis [24–26]. These target genes shared one or two common regions of miR-33b seeding sequences. Realtime PCR was performed to confirm the computational prediction results of these genes in Lu1205 and A375 cell lines. The cordycepin treatment downregulated the expression of *ZEB1*, *HMGA2* and *TWIST1* by more than 50% but had minimal effects on *ADAM9*, *RAC1*, *SALL4*, *CTNNB1*, *ZEB2* and *YES1* in these two melanoma cell lines (Figure 2A). Then, we cloned each 3′UTR of these 3 genes into pGL3-vector and conducted dual luciferase reporter assay to investigate whether miR-33b could directly regulate the expressions of these genes. 200 μg/ml cordycepin treatment and ectopic expression of miR-33b mimic dramatically reduced the luciferase activities of HMGA2, ZEB1 and Twist1 to the same extent (Figure 2B). This implied that cordycepin may target HMGA2, ZEB1 and Twist1 gene expression via miR-33b. To further validate the regulatory role of miR-33b, we inhibited miR-33b with lentivirus-based antagomir expression system to specifically knockdown precursor miR-33b (pre-miR-33b) and mature miR-33b in Lu1205 cells. The antagomir pairs reduced miR-33b levels in the context of cordycepin treatment by approximately 80% (data not shown). Then, we analyzed the effect of miR-33b knockdown on ZEB1, HMGA2 and Twist1 3′UTR activity. Knockdown of pre-miR-33b or miR-33b in Lu1205 cells rescued relative luciferase activities of ZEB1, HMGA2 and Twist1 3′UTR which have been suppressed by cordycepin treatment (Figure 2C). HMGA2, Twist1 and ZEB1 3′UTR activities were increased by 1.9-fold, 1.6-fold and 3.1-fold in Lu1205/cordycepin/sh-miR-33b cells, respectively. Knockdown of miR-33b in untreated cells did not significantly upregulate HMGA2, Twist1 and ZEB1 3′UTR activities.

To further determine whether miR-33b could regulate the expressions of these genes by directly binding to miR-responsive elements in 3′UTR, we analyzed the miR-33b binding sites in these genes' 3′UTR. There were two putative miR-33b binding sites in HMGA2 and one putative binding site in Twist1 and ZEB1. Quickchange PCR was employed to change the miR-33b seeding sequences in 3′UTRs of HMGA2, Twist1 and ZEB1. For HMGA2, two mutated luciferase reporters were generated by mutation of either binding site 1 (HMGA2-1 mut) or 2 (HMGA2-2 mut). Mutations of either binding site partially rescued the cordycepin-mediated decrease in luciferase activity, while simultaneous mutation of two binding sites completely rescued the suppressive effect of cordycepin (Figure 2D). Likewise, disruption of miR-33b binding regions in Twist1 and ZEB1 prevented the reporter from cordycepin-mediated downregulation of luciferase activity. All data suggest that HMGA2, Twist1 and ZEB1 are the downstream targets of miR-33b.

### miR-33b seed in melanoma patients was negatively correlated with HMGA2, Twist1 and ZEB1 expression levels

**Figure 3:**
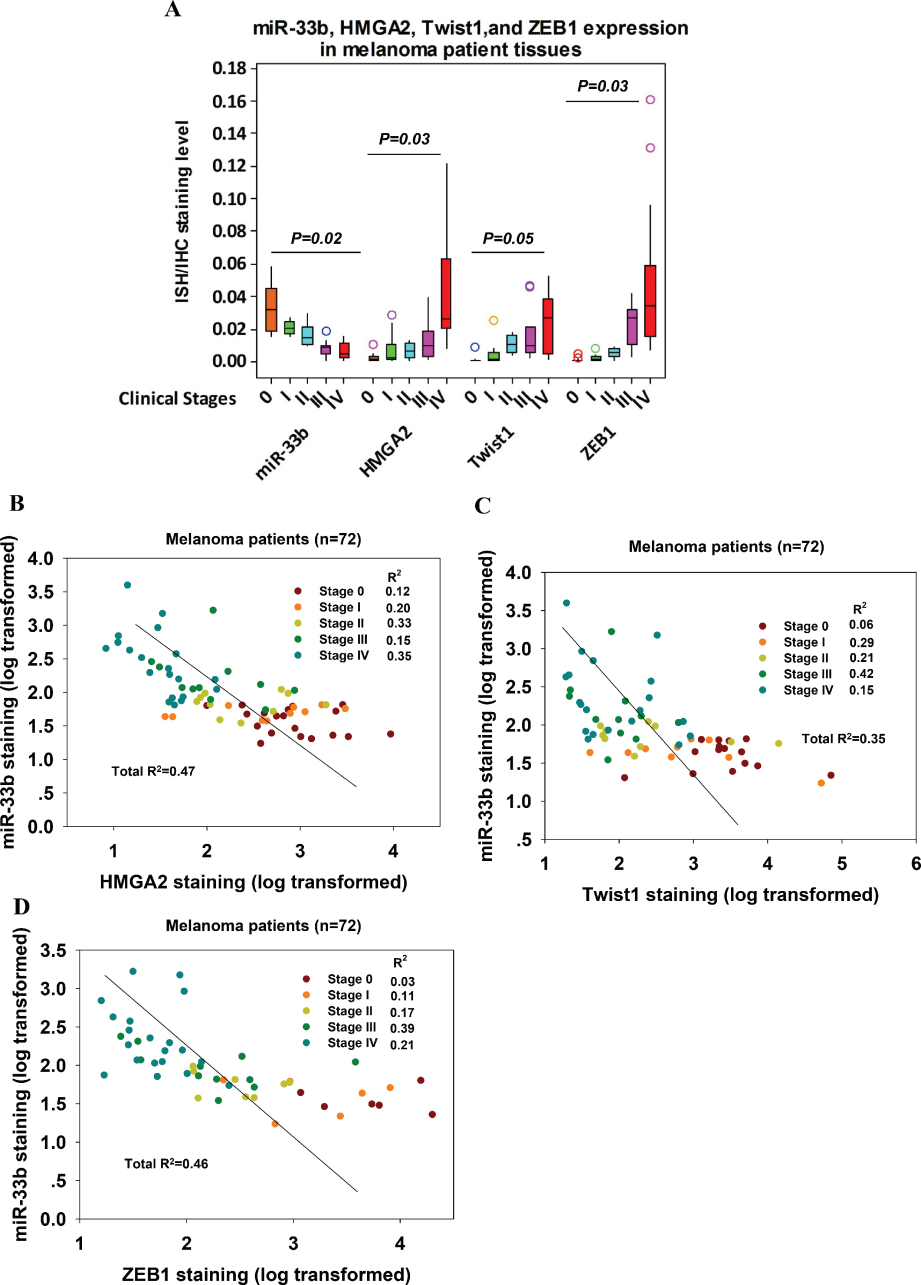
miR-33b levels in melanoma patient tissue samples were negatively correlated with those of HMGA2, Twist1 and ZEB1 **(A)** Relationship of miR-33b, HMGA2, Twist1 and ZEB1 relative expression and melanoma clinicopathological features. miR-33b expression levels in FFPE melanoma tissue samples from 72 patients with clinical TNM stage 0–IV were detected with *in situ* hybridization and quantified by ImageJ for grey value. HMGA2, Twist1 and ZEB1 expression levels in these FFPE melanoma tissue samples were detected with IHC and quantified by ImageJ for grey value. Relative expression levels of target molecules were compared among different TNM stages with ANOVA. *P* value is shown. Color circles represent outliers in each group. **(B–D)** The correlation between miR-33b relative expression levels (log transformed) and HMGA2 (B), Twist1 (C) or ZEB1 (D) relative expression levels (log transformed) in FFPE melanoma tissue samples from 72 patients with clinical TNM stage 0–IV. Pearson correlation coefficients and R^2^-values were calculated as indicated.

To assess whether there is an association between the levels of HMGA2, Twist1 or ZEB1 and miR-33b in melanoma, we analyzed their levels in 72 melanoma patient samples. The 72 melanoma patient samples were categorized according to their clinical stages (TNM) with stage 0 being the carcinoma *in situ* and stage IV being metastatic cancer. miR-33b levels were detected with *in situ* hybridization and HMGA2, Twist1 and ZEB1 expression levels were assessed with immunohistochemistry. miR-33b was negatively correlated with the grading of malignancy of melanoma (Figure 3A). Patients whose tumors express low miR-33b levels had reduced metastasis and relapse-free survival. HMGA2, Twist1 and ZEB1 expressions were significantly higher in samples with poor prognosis with stage IV tumor expressing highest levels of HMGA2, Twist1 and ZEB1. Using linear regression analysis, miR-33b displayed a significant negative correlation with the expressions of HMGA2, Twist1 and ZEB1, consistent with these genes being regulated by miR-33b in melanoma (Figure 3B–3D). Altogether, these data suggest that the loss of miR-33b leading to the upregulation of HMGA2, Twist1 and ZEB1 expressions may contribute to metastasis.

### Cordycepin suppresses HMGA2 and Twist1-mediated melanoma migration by targeting miR-33b

**Figure 4:**
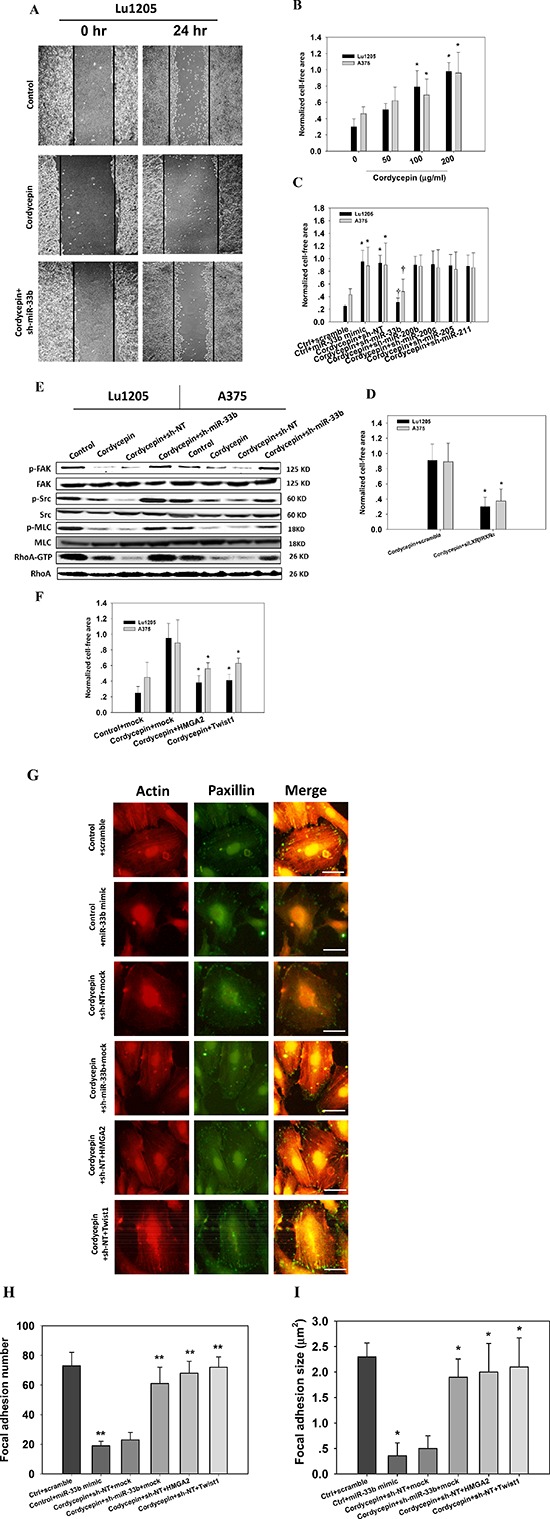
Cordycepin suppresses melanoma migration through miR-33b **(A–C)** sh-miR-33b reverted cordycepin-mediated suppression of Lu1205 melanoma migration capacities as measured by wound healing assay. (A) Representative images photographed at 0 and 24 hr post-wounding were shown at magnification of 20X. (B) Increasing cordycepin concentration reduced Lu1205 and A375 cell motility. (C) sh-miR-33b but not sh-miR-200b, sh-miR-200c, sh-miR-205 and sh-miR-211 reverted cordycepin-mediated suppression of Lu1205 and A375 motility. The cell free area were quantified at 24 hr and normalized against that at 0 hr. Data are expressed as mean ± SEM from three independent experiments. *p < 0.05 compared with control. ^†^*p* < 0.05 compared with sh-NT. **(D)** siLXRβ/RXRα cordycepin-mediated suppression of Lu1205 and A375 motility. The cell free area were quantified at 24 hr and normalized against that at 0 hr. Data are expressed as mean ± SEM from three independent experiments. *p < 0.05 compared with control. **(E)** sh-miR-33b reverted cordycepin-mediated suppression of phosphorylation of FAK, Src and MLC and GTP-coupling of RhoA in Lu1205 and A375 cells. Cells were untransfected or transfected with sh-NT or sh-miR-33b for 24 hr before being treated or untreated with 200 μg/ml cordycepin for 24 hr. Cells were lysed and subjected to Western blotting analysis of phosphorylation of FAK, Src and MLC and GTP-coupling of RhoA. Total FAK, Src, MLC and RhoA were used as loading controls. **(F)** HMGA2 and Twist1 overexpression reverted cordycepin-mediated suppression of Lu1205 migration as measured by wound healing assay. Lu1205 monolayer were transfected with either mock, HMGA2 or Twist1 construct before being treated with 200 μg/ml cordycepin for 24 hr. The cell free area were quantified at 24 hr and normalized against that at 0 hr. Data are expressed as mean ± SEM from three independent experiments. *p < 0.05 compared with cordycepin + mock. **(G)** Cordycepin inhibited Lu1205 melanoma stress fiber and focal adhesion formation via miR-33b. Lu1205 cells were transfected with a variety of miRNA mimic, sh-miRNA, and/or overexpressing constructs before being treated or untreated with cordycepin. Lu1205 cells were stained with rhodamine-phalloidin and paxillin antibody (Geen = paxillin, red = F-actin). Bar = 10 μm. **(H–I)** Quantification of the average number and size (μm2) of paxillin-containing focal adhesions in Lu1205 cells using ImageJ software.12 cells were analyzed per condition in each experiment. Data are expressed as mean ± SEM of three independent experiments. *p < 0.05, **p < 0.01 compared with ctrl + scramble.

Twist1 has been shown to mediate cancer migration through triggering Rac1 activation [27]. Twist also enhanced hepatocellular carcinoma EMT and invasion through activating matrix metalloproteinase (MMP)-2 and MMP-9 expression [28]. HMGA2 plays important roles in promoting cancer metastasis by downregulating miR-200b expression and increasing the level of lysyl oxidase (LOX) [29–31]. Therefore, we asked whether cordycepin can inhibit melanoma migration by targeting miR-33b and downregulating HMGA2 and Twist1 expression. We employed wound healing assays to assess the melanoma migratory capacities. 200 μg/ml cordycepin treatment inhibited the 24 hr wound closure capacities of melanoma cells (Figure 4A). sh-miR-33b transfection abrogated the suppressive effect of cordycepin on cell motility. Melanoma cells briskly migrated into the wound area with 24 hr after wound scratch. Quantitative analysis of the gap closure suggests that cordycepin significantly attenuated gap closure capacities of Lu1205 and A375 cells in a dose-dependent manner (*p* < 0.05) (Figure 4B). Ectopic expression of miR-33b significantly increased the cell-free gap area for Lu1205 and A375 cell lines (Figure 4C). In the context of cordycepin treatment, knockdown of miR-33b promoted the wound healing capacities (Figure 4C). The wound area in cordycepin/sh-miR-33b group reached 70% and 50% sealing for Lu1205 and A375 after 24 hr of wound scratch. In sharp contrast, miR-200b, miR-200c, miR-205 and miR-211 knockdown did not significantly alter the motility of melanoma, suggesting that miR-33b would be the sole miRNA responsible for cordycepin-mediated suppression of melanoma migration (Figure 4C). Silencing LXRβ/RXRα significantly ameliorated cordycepin-mediated inhibition of wound healing capacities (*p* < 0.05) (Figure 4D). Focal adhesion kinase (FAK), Src, myosin light chain (MLC) and RhoA activations are important for cancer motility. Cordycepin treatment suppressed the phosphorylation of FAK, Src and MLC and GTP-coupling of RhoA without affecting the total FAK, Src, MLC and RhoA expressions (Figure 4E). Knockdown of miR-33b in cordycepin-treated Lu1205 and A375 melanoma cells increased the levels of phosphorylated FAK, Src, and MLC and RhoA-GTP. This implies that cordycepin downregulates cell migratory machinery and cytoskeletal remodeling by targeting miR-33b. To determine whether the phenotypes related to cordycepin treatment can be reversed by restoration of HMGA2 and Twist1, we transfected cells with either mock (vector control), HMGA2 or Twist1 overexpression plasmids. HMGA2 or Twist1 overexpression reverted cordycepin-mediated reduction of melanoma motility (Figure 4F). They reduced the 24-hr cell-free area by 30%–55% in cordycepin-treated A375 and Lu1205 monolayer.

Since cytoskeletal structure may play important roles in cell migration, we analyzed the dynamics of actin cytoskeleton and focal adhesion. Migrating Lu1205 cells exhibited stress fiber formation with thick actin bundles traversing the cell bodies (Figure 4G). In contrast, ectopic expression of miR-33b or cordycepin treatment abolished the stress fiber structure in Lu1205 cells. miR-33b silencing, or overexpressing HMGA2 or Twist1 in cordycepin-treated Lu1205 cells restored actin polymerization. The stress fibers became more robust and organized. Paxillin is a multidomain scaffold adaptor protein which recruits structural and signaling molecules to focal adhesions. It plays an important role in transducing signals for cell adhesion and cytoskeletal reorganization [32]. By staining paxillin, it was shown that control cells displayed bright punctate focal adhesions which were colocalized with the end of thick stress fibers. However, in miR-33b mimic-transfected or cordycepin-treated cells, small focal adhesions were only visible at cell periphery and almost disengaged with thin stress fibers. miR-33b silencing or overexpressing HMGA2 or Twist1 reverted the suppressive effect of cordycepin on focal adhesion formation. Quantitative analysis revealed that cordycepin incubation decreased the size and number of focal adhesions via miR-33b, which could be rescued by restoring HMGA2 or Twist1 expressions (Figure 4H–4I). These data implied that focal adhesion assembly and cytoskeletal rearrangement were located in the downstream of HMGA2 and Twist1 whose expressions were diminished by cordycepin-induced miR-33b expression.

### Cordycepin inhibits HMGA2 and Twist1-mediated melanoma invasion via miR-33b

**Figure 5:**
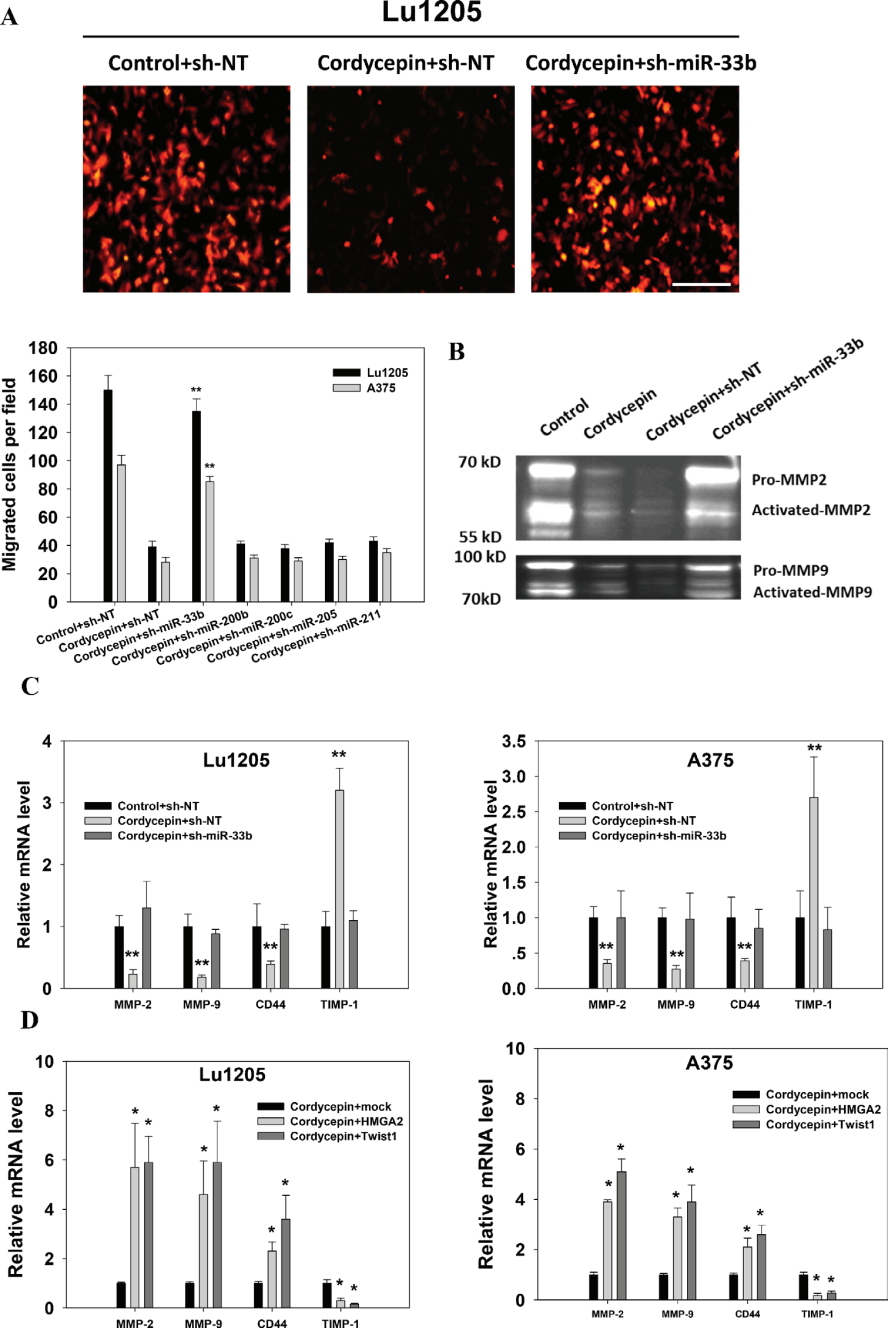
Cordycepin suppresses melanoma invasion through miR-33b **(A)** Matrigel-coated transwell invasion assay revealed that the miR-33b knockdown rescued cordycepin-mediated inhibition of Lu1205 and A375 invasion. *Top*, representative images show migrated RFP-Lu1205 cells underneath membrane. Bar = 100 μm. *Bottom*, quantification of number of migrated Lu1205 cells which were transfected with sh-NT or sh-miR-33b before being treated or untreated with cordycepin. Data are expressed as mean ± SEM from three independent experiments. **p < 0.01 compared with control+sh-NT. **(B)** Cordycepin downregulated MMP-2 and MMP-9 activities through miR-33b in Lu1205 cells as shown by gelatin zymograph assay. **(C)** qRT-PCR showed that cordycepin inhibited the mRNA expressions of invasion-related genes, MMP-2, MMP-9, and CD44, and increased the expression of TIMP-1 in Lu1205 and A375 cells. **(D)** Twist1 and HMGA2 restoration rescued cordycepin-mediated downregulation of mRNA expressions of invasion-related genes, MMP-2, MMP-9, and CD44, and decreased the expression of TIMP-1 in Lu1205 and A375 cells. Data are expressed as mean ± SEM from three independent experiments. *p < 0.05, **p < 0.01 compared with control+sh-NT or cordycepin+mock.

By degrading extracellular matrix, tumor invades underlying tissue and undergoes metastasis [33]. To determine whether miR-33b plays roles in cordycepin-mediated suppression of melanoma invasiveness, a Matrigel-based invasion assay was employed. While cordycepin significantly reduced Lu1205 and A375 invasiveness (*p* < 0.01), miR-33b knockdown rescued the cell invasion suppressed by cordycepin (Figure 5A). miR-200b, miR-200c, miR-205 or miR-211 knockdown did not change cell invasiveness. MMP-2/9 has been implicated in tumor invasion and metastasis [34–36]. We observed that cordycepin treatment resulted in reduced levels of pro- and cleaved-MMP-2 and MMP-9 as assessed by gelatin zymography assay (Figure 5B). The suppressive effect of cordycepin on MMP-2/-9 activities could be reverted by miR-33b antagomir. In addition, miR-33b antagomir attenuated the inhibitory effect of cordycepin on the expressions of MMP-2, MMP-9 and CD44 which are associated with tumor invasiveness at mRNA levels (Figure 5C). MMPs are under control of specific tissue inhibitors of metalloproteinases (TIMPs) which bind to catalytic domains of MMPs to inhibit their functions [36]. Among TIMPs, TIMP-1 specifically binds to pro-MMP-9 and inhibits MMP-9 function. We found that cordycepin treatment enhanced the expression of TIMP-1 and miR-33b knockdown attenuated TIMP-1 upregulation in both Lu1205 and A375 cells (Figure 5C). To determine whether changes of these gene expressions were dependent on the activities of HMGA2 and Twist1, we transfected melanoma cells with mock, HMGA2 or Twist1 constructs. In cordycepin-treated Lu1205 and A375 cells, HMGA2 and Twist1 overexpression significantly increased MMP-2, MMP-9 and CD44 but decreased TIMP-1 expression compared with mock (Figure 5D).

### miR-33b targets ZEB1 to regulate EMT following cordycepin treatment

**Figure 6:**
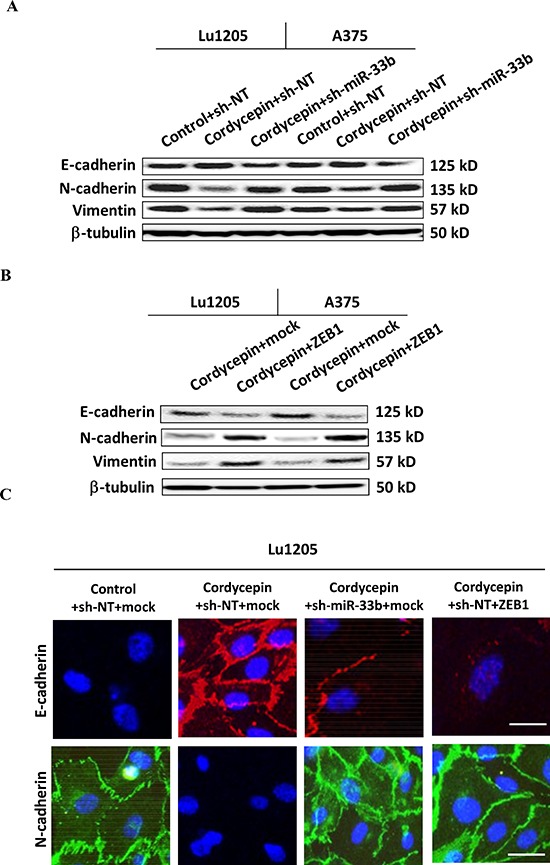
Knockdown of miR-33b promoted epithelial-mesenchymal transitions (EMT) of cordycepin-treated Lu1205 and A375 cells and re-expression of ZEB1 reversed miR-33b-dependent MET phenotype of Lu1205 cells **(A)** sh-miR-33b transfection or **(B)** ZEB1 overexpression reverted cordycepin-mediate MET in both Lu1205 and A375 cells as measured with Western blotting. E-cadherin is epithelial marker, while N-cadherin and vimentin are mesenchymal markers. β-tubulin serves as loading controls. **(C)** Immunofluorescence staining of E-cadherin (red) and N-cadherin (green) in untreated or cordycepin-treated Lu1205 cells which were transfected with sh-NT or sh-miR-33b and mock or ZEB1. Nuclei were counterstained with DAPI. Bar = 10 μm.

EMT plays important roles in melanoma metastasis [37]. Cordycepin treatment reduced the expression of mesenchymal markers, N-cadherin and vimentin, and upregulated the expression of epithelial protein, E-cadherin (Figure 6A). In contrast, miR-33b knockdown reverted the trend and promoted mesenchymal differentiation of Lu1205 and A375 cells. To determine the roles of ZEB1 which is a critical mediator of EMT during cancer progression [25, 26], we transfected cells with mock and ZEB1 constructs. Overexpression of ZEB1 elevated the expression of N-cadherin and vimentin but suppressed the expression of E-cadherin (Figure 6B). In agreement with western blotting results, immunofluorescence staining suggested that miR-33b silencing or ZEB1 overexpressing reverted cordycepin-mediated epithelial differentiation of melanoma cells (Figure 6C).

### Cordycepin targets miR-33b to inhibit orthotopic and experimental melanoma metastasis to multiple organs *in vivo*

**Figure 7:**
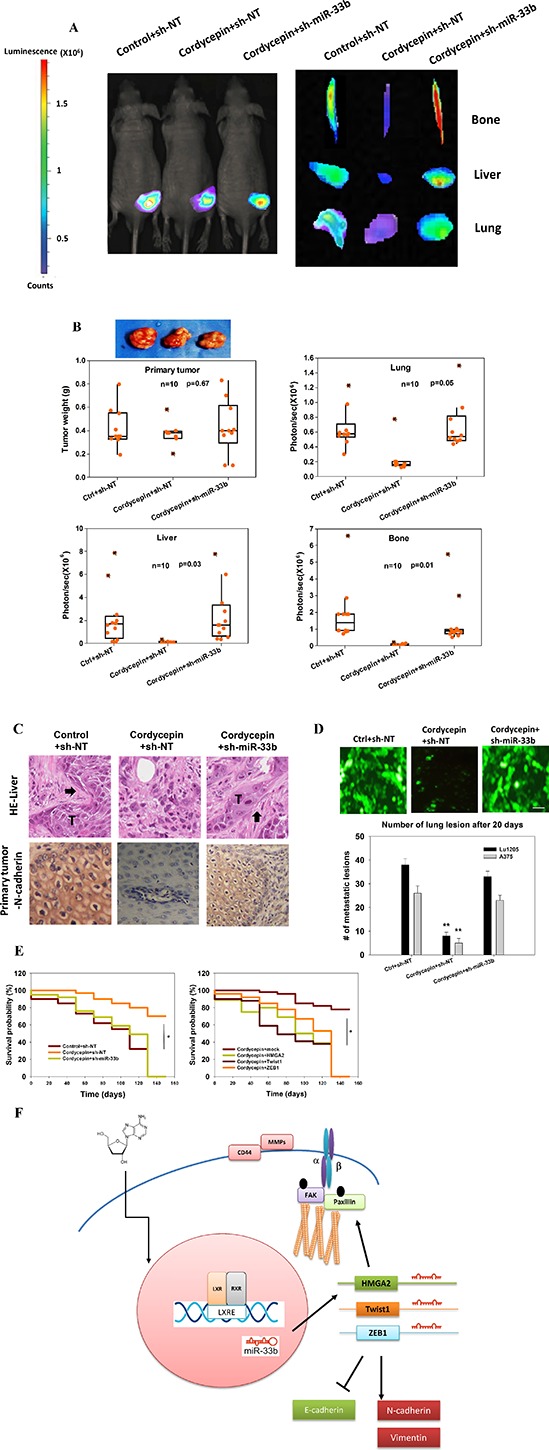
Cordycepin suppresses spontaneous metastasis through miR-33b **(A)** Bioluminescence images of primary tumor, lung, liver and bone from sh-NT or sh-miR-33b stable Lu1205 cells following 28 days post implantation. **(B)** Quantification of primary tumor weight (g) and metastasis to the lung, liver and bone as measured by bioluminescent intensity (represented by photons/sec). Cordycepin (3′-deoxyadenosine) was administrated i.p. daily to the nude mice in a dosage of 2 mg/kg body weight after the tumor inoculation. Ten mice were injected under each condition with each point representing a measurement from an individual mouse with the quartiles and median values indicated by a boxplot. *P*-values were calculated using a two-tailed Mann-Whitney *U*-test. **(C)** H&E (upper) staining of liver tissue and IHC (lower) staining of N-catenin in primary tumor. Arrow: metastatic tumor. **(D)** Prominent lung lesions of injected metastatic GFP-Lu1205 and GFP-A375 cells transfected with sh-NT and sh-miR-33b. Vesicle (ctrl) or cordycepin was administrated one week before tumor inoculation. Images were representative of six independent experiments. Number of lung lesions was quantified. **p < 0.01 compared with control. **(E)** Kaplan–Meier analysis comparing survival in sh-NT- and sh-miR-33b-transfected mice (left) and vector, HMGA2, Twist1 and ZEB1-transfected mice (right). Statistic significance between groups was analyzed by log-rank test. *p < 0.05 among groups. **(F)** Model of melanoma cancer metastasis suppressed via cordycepin-initiated signaling pathways.

To determine whether cordycepin influences spontaneous melanoma metastasis, we implanted nontargeting control (sh-NT) or sh-miR-33b-expressing Lu1205 cells to the right flank of nude mice and determined the primary tumor growth and metastasis to multiple organs by bioluminescence imaging. The 28-day tumor weight and size were comparable for control+sh-NT, cordycepin+sh-NT and cordycepin+sh-miR-33b groups (Figure 7A–7B). Nevertheless, cordycepin-treated melanoma metastasized less efficiently to lung, liver and bone. miR-33b knockdown in melanoma cells rescued the suppressive effect of cordycepin on metastasis (Figure 7A–7B). Cordycepin treatment or miR-33b knockdown did not affect the growth of the primary tumors, but did reduce the metastatic potential of cells in the spontaneous metastasis assay, therefore, indicating the involvement of miR-33b in the cordycepin-regulated melanoma invasiveness. To examine whether reduced metastasis of melanoma was due to the fact that primary tumor has underwent an epithelial differentiation, we stained primary tumor for N-cadherin. H&E staining verified bioluminescence results for the involvement of miR-33b in cordycepin-regulated melanoma liver metastasis (Figure 7C). N-cadherin expression was weak in primary tumor which was transfected by sh-NT and treated with cordycepin in comparison with vesicle-treated or cordycepin-treated/sh-miR-33b-transfected melanoma cells. miR-33b knockdown restored the robust N-cadherin staining in the cell membrane and cytosol of primary tumor in response to cordycepin treatment. This suggested that mesenchymal-epithelial transition (MET) was necessary for miR-33b-suppressed melanoma metastasis. Metastasis is a multistep process involving the early steps of EMT and intravasation followed by extravasation and colonization. To circumvent the early step of metastasis and specifically investigate whether miR-33b inhibits melanoma extravasation and colonization, we injected sh-NT or sh-miR-33b-expressing GFP-tagged melanoma cells via tail vein and monitored lung metastasis. In vesicle+sh-NT and cordycepin+sh-miR-33b cohorts, cells were completely cleared from the circulation in lung, 48-hr after tail vein injection. On the contrary, GFP-Lu1205 or GFP-A375 cells were trapped in the lung vein in cordycepin+sh-NT group, suggesting that cordycepin reduced cell extravasation by targeting miR-33b. After 20 days, appreciable amounts of tumor lesions formed in lung in control group (Figure 7D). Cordycepin treatment reduced the number of metastatic nodules in the lung of mice receiving Lu1205 and A375 cells by 78% and 80%, respectively. miR-33b knockdown rescued the abilities of Lu1205 and A375 cells to extravasate and colonize in the lung. Thus, miR-33b upregulated by cordycepin not only inhibited early stages of metastasis, but also repressed cell migration and invasion. The survival time of mice given cordycepin was significantly longer than that of control mice (Figure 7E). In contrast, miR-33b knockdown or HMGA2, Twist1 or ZEB1 overexpression significantly attenuated the effect of cordycepin on lifespan of tumor-bearing mice.

## DISCUSSION

With its low toxicity towards normal cells, cordycepin has long been known to act as a therapeutic or preventative agent for several tumor cells [5, 6]. Uncertainty regarding the multiple anti-metastatic functions of cordycepin necessitates the studies like the current one to dissect its various roles, which seems to be dependent on tumor stages, molecular interactions and transcriptional regulations. In the current study, we found that cordycepin could suppress melanoma invasion via MMPs and metastasis via actomyosin machinery through LXR/RXR activation-dependent upregulation of miR-33b (Figure 7F). Cordycepin suppressed HMGA2, Twist1 and ZEB1 expressions through miR-33b. Upregulation of miR-33b by cordycepin further resulted in MET and inhibited melanoma metastasis *in vivo*. Our concepts strengthen the potential of cordycepin as a multitarget drug in anti- metastatic therapy.

Previous studies suggest that cordycepin inhibits MMP-2 and MMP-9 expression and suppresses MAPK/AP-1 and Akt/PI3K pathways in breast cancer and prostate carcinoma cells, suppressing cancer invasiveness [7, 38]. In addition, cordycepin has been shown to suppress the expression of E-cadherin and integrins, the partners of FAK, in regulating the focal adhesion complex and preventing EMT in hepatocellular carcinoma [39]. These are in consistent with our findings that cordycepin suppresses cancer metastasis through modulating the focal adhesion dynamics and inhibiting extracellular matrix breakdown. Some studies reported that cordycepin could induce cancer G2/M arrest via p21WAF1 expression and promote cancer apoptosis [6, 40]. In the current study, we did not observe a prominent anti-proliferative and pro-apoptotic effect of up to 200 μg/ml cordycepin treatment on melanoma cells with MTT assay (data not shown) and *in vivo* primary tumor growth (Figure 7A). Therefore, cordycepin effect on tumor progression may be dependent on cancer type, stage and dosage.

The emerging concept of comparing miRNA expression profiles after small compound treatment of specific diseases has been indicated to be an effective way of identifying new drug targets [41]. By analyzing miRNA array for miRNA differentially expressed from different stages of melanoma, miR-203, miR-205 and miR-211 were identified as the targets [42]. miR-203, miR-205 and miR-211 were proposed to act as tumor suppressors to limit anchorage-independent growth. In agreement with this, we identified miR-205 and miR-211 as cordycepin-upregulated tumor suppressive miRNAs in our miRNAarray data set. However, knockdown of miR-200b, miR-200c, miR-205 and miR-211 did not affect cordycepin-mediated suppression of melanoma migration and invasiveness (Figure 4C and Figure 5A). Given that miR-200 family can suppress EMT by repressing ZEB1 expression, it was likely that some inhibitors for accessibility of mRNA to miRNA or the local secondary conformation of mRNA might disrupt the targeting abilities of these miRNAs [43, 44]. In this study, we reported that miR-33b was upregulated by cordycepin and miR-33b expression was negatively correlated with clinical stages of melanoma. In addition, we found that miR-33b represses *in vivo* melanoma lung, liver and bone metastasis. These data may imply that miR-33b is not only a sensor for cordycepin treatment but also functions as tumor-suppressor miRNA to have prognostic significance. Of note, previous studies indicated that miR-33b expression was inversely correlated with malignancy of human breast cancer [45]. Therefore, miR-33b may be a marker to classify the metastatic stages. miR-33b is located in intron 17 of the endoplasmic reticulum (ER)-bound sterol regulatory element-binding protein-1 (SREBP-1) gene on chromosome 17 [46]. miR-33 is co-transcribed with SREBP genes, reducing cholesterol export [21, 46]. miR-33 also binds to 3′UTR of carnitine O-octaniltransferase (CROT), Carnitine palmitoyltransferase 1A (CPT1a) and hydroxyacyl-CoA-dehydrogenase (HADHB) to regulate fatty acid metabolism [47]. A recent study suggests that another membrane of miR-33 family, miR-33a, enhances glioma-initiating cell self-renewal through PKA and NOTCH pathways [48]. In addition, miR-33a was implicated in mediating anti-apoptotic effect on cisplatin-resistant osteosarcoma by targeting TWIST [49]. It was reported that the expressions of miR-33a and miR-33b were independently regulated in that miR-33a expression was controlled by SREBP-2, while miR-33b expression was triggered by LXR [21]. In the current study, we found that cordycepin induces the binding of LXR/RXR to LXRE of SREBP-1 promoter and silencing LXRβ/RXRα abrogated cordycepin-induced miR-33b expression. Further studies were undertaken to elucidate whether cordycepin may enhance the binding of activator to LXR/RXR or alleviate the repression of LXR/RXR. The regulation of downstream gene expression by miR-33b diverges from the well-established induction of genes related to lipid metabolism. Cordycepin may initiate distinct signaling pathways to induce miR-33b expression and prescribe the downstream targets of miR-33b. We speculate that the target gene transcription regulation abilities of miR-33b are largely dependent on cellular environment. Indeed, it was reported that RNA-binding factors may inhibit miRNA access to certain target mRNA [44].

HMGA2 belongs to high motility group A family, being a non-histone chromatin protein which alters chromatin structure and regulate the transcription of several genes [50]. HMGA2 expression is correlated with the presence of metastasis and a reduced survival. HMGA2 gene was also found to be a target of chromosomal translocations, resulting in generation of fusion genes encoding a truncated HMGA2 protein that loses its carboxyl terminus, including 3′UTR. Since 3′UTR facilitates miRNA binding, truncating the HMGA2 3′UTR may abrogate the negative regulation by miRNA [50]. HMGA2 has been suggested to mediate breast tumor metastasis by promoting the expression of LOX and syndecan-2 [30]. Previous evidence also indicated that HMGA2 is fused to LIM domain containing preferred translocation partner in lipomas (LPP) gene and localized to focal adhesions as well as cell-to-cell contacts [50]. In the current study, we demonstrated that HMGA2 was involved in cordycepin-mediated suppression of late-stage melanoma metastasis through modulation of the activation status of FAK, Src, MLC and RhoA and expression of MMPs. Transcriptional factor, Twist1, was shown to regulate cancer cell migration and invasion [51]. Twist was suggested to be targeted by miR-720 in breast cancer to promote tumor metastasis [52]. Overexpression of Twist1 in melanoma augmented melanoma migration and invasion via MMP-1 [53]. Interestingly, Twist1 can elicits cancer movement through activation of Rac1 GTPase [27]. Herein, we report that cordycepin might regulate focal adhesion assembly and MMP expression through modulating the expression of the Twist1 gene via miR-33b. Since HMGA2 and Twist1 are transcriptional regulators, more studies are needed to clarify whether HMGA2 and Twist1 directly or indirectly regulate cell motility and expression of MMPs and CD44 in response to cordycepin treatment and miR-33b upregulation. In the current study, we showed that miR-33b by binding to 3′UTR of ZEB1 to suppress EMT process. Previously, ZEB1 has been suggested to be the primary EMT mediator in multiple cancer types [54]. ZEB1 triggers a miR-200 family-mediated negative feedback loop that stabilizes EMT and promotes invasion of cancer cells [25, 26]. We demonstrated for the first time that miR-33b can suppress EMT through modulating the expression ZEB1. Although it was reported that other transcriptional regulator, like HMGA2, can stimulate EMT, ZEB1 may function as the predominant trigger for EMT in melanoma cells, as overexpression of ZEB1 in melanoma cell was sufficient to revert cordycepin-mediated inhibition of EMT (Figure 6).

In conclusion, we provide strong evidence supporting the idea that cordycepin is able to inhibit the expression of HMGA2, Twist1 and ZEB1 by targeting miR-33b. This indicates that the anti-invasive and anti-metastasis effects of cordycepin are likely to be mediated through the induction of miRNA. In addition, cordycepin was able to decrease the expression of MMP-2, MMP-9 and CD44 and suppress cell motility, suggesting that cordycepin not only regulates early stages of melanoma metastasis, but also late stages of melanoma dissemination, such as adhesion and extravasation. The miRNA-targeting mechanism of cordycepin may provide a new perspective of how cordycepin can be used in treatment of metastatic melanoma. Our findings help to shed light on the development of new drugs such as cordycepin analogues that may inhibit cancer metastasis by targeting miRNA.

## MATERIALS AND METHODS

### Primary human melanoma tissue samples

Tissue collection and analysis in this study were approved by the Ethics Committee of Third Military Medical University of China, and written informed consent to perform the biological studies obtained from all participants. Formalin-fixed paraffin-embedded tissue (FFPE) sections of skin tumor of 72 melanoma patients were collected. The 72 melanoma patients consist of 30 females and 42 males, ages 18 to 83 years (median, 53 years). 21 patients had clinical stage 0 disease, 10 patients had stage I disease, 10 patients had stage II disease, 11 patients had stage III disease, and 20 patients had stage IV disease. Cases were selected from original hematoxylin and eosin (H&E) stainings. The differences of variables, like age and sex, among TNM stages are statistically insignificant.

### Reagents and cell culture

Cordycepin was purchased from Sigma-Aldrich (Saint Louis, MO). The Lu1205, A375, GFP-tagged Lu1205, GFP-tagged A375, RFP-tagged Lu1205 and RFP-tagged A375 melanoma cell lines (obtained from ATCC) were maintained in Dulbecco's modified Eagle's medium (DMEM; GIBCO) supplemented with 10% FBS and 100 U/ml of penicillin-streptomycin. All cells were maintained in a humidified incubator at 37°C and 5% CO_2_.

### microRNA array

Total RNA was isolated from melanoma cells using RNeasy Mini Kit (Qiagen) according to the manufacturer's instructions. The quality and quantity of RNA samples were assessed by standard electrophoresis. The array hybridization on a miRCURY LNA microRNA array (v.10.0) (Exiqon Life Sciences, Vedbaek, Denmark) was carried out. Each microarray chip was labeled with either Hy3^TM^ or Hy5^TM^, and hybridized to the array slide. The arrays were scanned and analyzed using GenePix Pro (Molecular Devices). The data were corrected for background and normalized. The normalized data was utilized to obtain values for changes of expressions among samples. miRNAs with > 2-fold change were designated as differentially expressed.

### Xenograft tumor model

All the procedures involving animals were reviewed and approved by Pennsylvania State University Institutional Animal Care and Use Committee. For orthotopic metastasis assay, five-week-old athymic nu/nu male Balb/c mice (Herlan, Indianapolis, IN) were implanted subcutaneously in the right flank with melanoma cells expressing miRNA antagomir or overexpression constructs (1 × 10^6^ cells/100 μl PBS/Matrigel). When tumors have reached 0.35 cm in diameter, the mice were randomized into the following treatment groups (*n* = 10/group): (i) untreated control (ethanol in PBS (10%; v/v), 100 μl intraperitoneally (i.p.)); (ii) cordycepin (2.0 mg/kg of body weight, suspended in ethanol in PBS (10%; v/v), i.p., daily. The treatment was continued for 4 weeks before sacrifice. The body weights and tumor sizes were recorded every 3 days, and the tumor size was determined by a Vernier caliper measurement. Bioluminescence imaging was performed. Primary tumors were excised and the tumor weights were measured. In selected cases, the tumor tissues were fixed in formalin and embedded in paraffin for immunohistochemistry and routine H&E staining. For experimental metastasis, 1 × 10^6^ GFP-Lu1205 or GFP-A375 cells were injected intravenously into nude mice. Mice were killed 20 days after cell injection and the presence of fluorescent metastatic lesions was detected using Nikon SMZ 1500 dissecting microscopy. 12 images of random fields were photographed at the magnification of X40 from each lung and the number of lesions was counted.

### Bioluminescence imaging

Mice were injected i.p. with 30 mg/ml of D-luciferin 10 min before imaging. Dorsal images of the primary tumors were collected before the mice were culled and their primary tumor, lung, liver and hind limbs were harvested for *ex vivo* imaging. Bioluminescence imaging was conducted with Xenogen IVIS 100 imaging system and photon emission was quantified with Xenogen software (Perkin Elmer, Hopkinton, MA).

### *In situ* hybridization and immunohistochemistry

For *in situ* hybridization (ISH), biotin-labeled probes were purchased from Exiqon for human miR-33b and a control scramble miRNA probe. Tumor tissue slides from melanoma patients were hybridized in 50 nM of probe diluted in 500 μL of hybridization buffer at 30°C. Hybridization was performed on a Hybridizer (Dako) overnight. Slides were then incubated in ExtrAvidin–alkaline phosphatase (Sigma), followed by incubation in detection buffer and then in BM Purple AP Substrate (Roche). ISH results were quantitatively measured with ImageJ and normalized against scramble control. For immunohistochemistry (IHC), FFPE from patient and mouse samples were incubated with anti-HMGA2 (Cell Signaling), Twist1 (Abcam), ZEB1 (Santa Cruz) or N-cadherin (Abcam). The target proteins were detected with 3,3′diaminobenzidine (DAB). Nuclei counterstained with hematoxylin. The gray values of staining were quantified with ImageJ.

### Transfection with miRNA mimics, miRNA hairpin inhibitors, and expression constructs

Transfections of miRNA mimics, miRNA antagomirs and cDNA constructs encoding ZEB1, Twist1 and HMGA2 were carried out using the Lipofectamine 2000 (Invitrogen, Carlsbad, CA) or Mirus transfection reagent. The detailed conditions are described in the Supplementary Materials.

### Dual-luciferase reporter assay

3′UTRs of the *TWIST1, ZEB1 and HMGA2* genes and LXRE fragments of human sterol regulatory element-binding protein-1 (SREBP-1) gene promoter were amplified by PCR using genomic DNA and were inserted into the XbaI and FseI sites of pGL3 control vector (Promega, Madison, WI). Luciferase assay was performed with assay kits (Promega). The detailed conditions are described in the Supplementary Materials.

### Quantitative RT-PCR of mRNAs and miRNAs

qRT-PCR was used to quantify miR-200b, miR-200c, miR-205, miR-33b, and miR-211 and mRNA expressions. For miRNAs, total RNA was reversed transcribed with stem-loop RT primers and measured with qRT-PCR kit. The detailed conditions and primers are described in the Supplementary Materials.

### Wound healing assay

Detailed procedure was described in the Supplementary Materials.

### Matrigel invasion assay

Cell invasiveness was reflected by the ability of cell to transmigrate through a layer of Matrigel in BioCoat Matrigel Invasion Chambers (8 μm pores) (BD Biosciences, Bedford, MA). The detailed conditions are described in the Supplementary Materials.

### Gelatin zymography

Gelatin zymography was used to detect MMP activity in melanoma cell. The detailed conditions are described in the Supplementary Materials.

### Western blotting

Detailed conditions for western blotting, including antibodies used, are described in Supplementary Materials.

### Immunofluorescence

The dynamics of focal adhesions, actin, and EMT markers were measured with immunofluorescence microscopy. The detailed conditions are described in the Supplementary Materials.

### Statistical analysis

Statistical significance of differences between means was determined by using a two-tailed Mann-Whitney *U* test if the distribution of the measurement is non-normal, Student's *t*-test if normality holds and there are two means for comparison, or analysis of variance (ANOVA) if there are more than two means. Tukey's test was used for *post hoc* analysis of ANOVA to find means that are significantly different from each other. Survival data was analyzed with the log-rank test. Probability values of *p* < 0.05 and *p* < 0.01 were considered to be statistical significant. The linear correlation was calculated based on the Pearson correlation coefficient.

## SUPPLEMENTARY MATERIALS AND METHODS


