# Supplementary material for: Cordycepin (3′-deoxyadenosine) suppressed HMGA2, Twist1 and ZEB1-dependent melanoma invasion and metastasis by targeting miR-33b
Source: Oncotarget. 2015 Mar 13;6(12):9834–53. doi: 10.18632/oncotarget.3383 (PMC4496401; doi:10.18632/oncotarget.3383)
Supplement: Supplementary file 1 [file oncotarget-06-9834-s001.pdf]

SUPPLEMENTARY MATERIALS AND METHODS

Primers for qRT-PCR

CD44: 5′-TGCCGCTTTGCAGGTGTAT-3′ (forward) and 5′-GGCCTCCGTCCGAGAGA-3′ (reverse);

ADAM9: 5′-CAGATGGCAAAAATCAAGCA-3′ (forward) and 5′-GATGGGAACTGCTGAGGTTG-3′ (reverse);

ZEB1: 5′-AAGAATTCACAGTGGAGAGAAGCCA-3′ (forward) and 5′-CGTTTCTTGCAGTTTGGGCATT-3′ (reverse);

ZEB2: 5′-CGCTTGACATCACTGAAGGA-3′ (forward) and 5′-CTTGCCACACTCTGTGCATT-3′ (reverse);

CTNNB1: 5′-GAAACGGCTTTCAGTTGAGC-3′ (forward) and 5′-CTGGCCATATCCACCAGAGT-3′ (reverse);

HMGA2: 5′-GCGCCTCAGAAGAGAGGAC-3′ (forward) and 5′-GGTCTCTTAGGAGAGGGCTCA-3′ (reverse);

RAC1: 5′-CTGCCAATGATATGGTAGATG-3′ (forward) and 5′-CCGCACCTCAGGATACCA-3′ (reverse);

SALL4: 5′-TGCAGCAGTTGGTGGAGAAC-3′ (forward) and 5′-TCGGTGGCAAATGAGACATTC-3′ (reverse);

Twist1: 5′-ACGAGCTGGACTCCAAGATG-3′ (forward) and 5′-CACGCCCTGTTTCTTTGAAT-3′ (reverse);

YES1: 5′-AAGCTGCACTGTATGGTCGGTTTA-3′ (forward) and 5′-GGGCACGGCATCCTGTATCCTC-3′ (reverse);

MMP2: 5′-GGCCCTGTCACTCCTGAGAT-3′ (forward) and 5′-GGCATCCAGGTTATCGGGGA-3′ (reverse);

MMP9: 5′-GGAGACCTGAGAACCAATCTC-3′ (forwards) and 5′-TCCAATAGGTGATGTTGTGGT-3′ (reverse)

TIMP1: 5′-CTGTTGTTGCTGTGGCTGATA-3′ (forwards) and 5′-CCGTCCACAAGCAATGAGT-3′ (reverse)

GAPDH: 5′-GACTCATGACCACAGTCCATGC-3′ (forward) and 5′-AGAGGCAGGGATGATGTTCTG-3′ (reverse).

Computational target prediction

For the identification of miR-33b targets, three different software algorithms were used to find conserved target sites throughout transcriptomes: TargetScan (http://www.targetscan.org), PicTar (http://pictar.mdc-berlin.de) and miRanda (http://www.microrna.org). The three lists obtained from *in silico* computational target predictions were compared. The intersection of resulted in a list of 10 common genes as potential targets.

Transfection with miRNA mimics, miRNA hairpin inhibitors, and expression constructs

Negative control, hsa-miR-33b mimic, siLXRβ, and/or siRXRα (siGENOME SMARTpool (M-003443-02)) (100 pmol) (Mission miRNA mimic, Sigma) was introduced into Lu1205 and A375 cells with 5 µl Lipofectamine 2000 in serum-free medium 24 hr after plating. For silencing pre-miR-33b, miR-33b, miR-200b, miR-200c, miR-205 and miR-211, sh-NT (nontargeting control), sh-pre-miR-33b (pre-miR33b antagomir), sh-miR-33b (miR-33b antagomir), sh-miR-200b (miR-200b antagomir), sh-miR-200c (miR-200c antagomir), sh-miR-205 (miR-205 antagomir) and sh-miR-211 (miR-211 antagomir) were synthesized following established protocol by RiboBio (RiboBio Co. Ltd, Guangzhou, China). (20). The short hairpin antagomirs were constructed and cloned into pTRIPZ empty vector, respectively (Openbiosystems, Thermofisher, Epsom, UK). Packaging was performed using a second generation plasmid system (psPAX2 and pMD2.G; Addgene, Cambridge, MA) by transient transfection of 293T cells. Then, the virus from the culture medium was concentrated and used to infect Lu1205 and A375 cells. Cells were infected 24 to 48 hr after transfection in the presence of polybrene (Sigma). The stable cell lines were selected in puromycin (Merck). To induce antagomir expression, cells were treated with 1 µg/ml of doxycyclin (Sigma).

The mimic sequences were: siLXRβ sense: 5′-AGAUCGUGGACUUCGCUAA-3′; miR-33b mimic sense: 5′-GUGCAUUGCUGUUGCAUUGC-3′; negative control sense (scramble): 5′-UUCUCCGAACGUGUCACGUTT-3′.

cDNA clones of human ZEB1, Twist1 and HMGA2 were obtained from Origene (Rockville, MD). The cDNA constructs were inserted into the expression vector pcDNA3.1 (Invitrogen). 200 ng constructs were transfected into Lu1205 and A375 cells with Mirus TransIT (Mirus Bio LLC.) and then subjected to G418 selection for 2–3 weeks. Expression of the transfected constructs was assessed by Western blot analysis.

Dual-luciferase reporter assay

The following primers were used to amplify Twist1, ZEB1 and HMGA2 from human genomic cDNA: Twist1: (sense) 5′-GCGCCTCGAGCAGGCGGAGCCCCCA

CCCCCTCA-3′ and (antisense) 5′-GCGCGCGG CCGCGCAGAAAAATAT ACAAAGATATT-3′; ZEB1:

(sense) 5′-ATAATACGCG TTAAAGGAAGCT GATTAATTAGATATGC-3′ and (antisense) 5′-ATAATA AGCTTTTTGTAGTGCAGAAGTT CTCACATTTT-3′; HMGA2: (sense) 5′-GAGGAAACTGAAGAGACATC CTC-3′; and (antisense) 5′-GTTAGAAGACACTCAAA GGAACAG-3′.

Mutant 3’UTR of these genes were generated by site-directed mutagenesis. For reporter assay, Lu1205 and A375 cells were plated onto 12-well plates and transfected with 100 ng of pGL3-3’-UTR of genes using Lipofectamine 2000 (Invitrogen). A Renilla luciferase vector pRL-SV50 (5 ng; Promega) was also co-transfected to normalize the differences in transfection efficiency. After transfection for 48 hr, cells were harvested and assayed with Dual-Luciferase Reporter Assay System (Promega) using a Tecan M200 luminescence reader according to the manufacturer’s instructions. Transfection was repeated three times in triplicate.

Chromatin immunoprecipitation assay

Lu1205 cells were treated with cordycepin before ChIP assay was conducted using ChIP-IT kit (Active Motif) according to manufacturer’s instructions. Briefly, cells were treated with 5% formaldehyde for 20 min to cross-link DNA with proteins, lysed, passed through a 26 gauge-needle to facilitate nuclei release, and sonicated. After pre-clearing the chromatin with protein G beads, the supernatants were immunoprecipitated with 1 μg anti-LXR antibody or 1 μg negative control IgG overnight. Then, the protein G-antibody-chromatin complex was washed and eluted from the protein G. DNA was purified with supplied columns and samples were analyzed by PCR.

Quantitative RT-PCR of mRNAs

Total RNA was isolated using the RNeasy mini (Qiagen), and cDNA was synthesized from mRNA using the PrimeScript™ II 1st Strand cDNA Synthesis Kit (Takara) according to the manufacturer’s instructions. For qPCR, the amount of mRNA was monitored with SYBR Green™ qPCR SuperMix reagent (Invitrogen) with primer pairs specific for CD44, ADMA9, ZEB1, ZEB2, CTNNB1, HMGA2, Rac1, SALL4, Twist1, Yes1, MMP-2, MMP-9 and TIMP-1. The reactions were performed in 96-well plate using a preheated real-time instrument (ABI 7900HT).

Quantitative RT-PCR of miRNAs

For miRNAs, the isolated total RNA was polyadenylated and reverse transcribed for use in a two-step quantitative RT-PCR using the TaqMan microRNA reverse transcription kit and qRT-PCR kits (Invitrogen) according to the manufacturer’s instructions. Primers specific for human, miR-200b, miR-200c, miR-205, miR-33b, and miR-211 (Qiagen) were used. The relative amounts of miRNA to small nuclear RNU6B RNA was calculated using equation $2^{-\Delta Ct}$, where $\Delta Ct = (Ct_{-miR} - Ct_{-RNU6B\ RNA})$. The PCR conditions were 2 min at 50°C and 10 min at 95°C, followed by 40 cycles of 95°C for 15 s and 60°C for 30 s.

Wound healing assay

One day before transfection, equal numbers of Lu1205 and A375 cells were seeded onto 24-well plates. Cells were then transfected with miR-33b antagomir, HMGA2 construct, Twist1 construct or controls. When the cell confluence reached about 90% after transfection, cell monolayer was treated or untreated with cordycepin at different doses for 24 hr. An artificial homogenous wound was made onto the monolayer with the use of a sterile plastic 200 μl micropipette tip. After wounding, the debris was removed by washing the cells with PBS. At different time points, cells that migrated into the wounded area were photographed under an inverted microscope (40× objective) (Nikon TE2000-U, Nikon Inc.). Wound healing effect was determined by calculating the ratio of the cell-free area at specific time point to that at time 0.

Matrigel invasion assay

RFP-Lu1205 and RFP-A375 cells were transfected with nontargeting control or miR-33b antagomir as mentioned above. Transfected cells (0.5×10^6) were reseeded into the rehydrated insert after treated with cordycepin. Medium with 10% FBS was added to the lower chamber as chemoattractant. After 24 hr incubation, non-invading cells on the upper surface of the membrane were scrubbed gently with a cotton-tipped swab. The invasive cells were photographed under an inverted light microscope (40 × objective) and quantified by manual counting in 6 randomly selected areas.

Gelatin zymography

Lu1205 and A375 cells were transfected with sh-NT or sh-miR-33b before being treated with cordycepin. After 24 hr, the medium that was collected from the incubated cells was mixed with substrate gel sample buffer (40% (v/v) glycerol, 0.25 M Tris-HCl, pH 6.8, and 0.1% bromophenol blue), and then it was loaded without boiling onto 10% SDS-polyacrylamide gel that contained gelatin (1.5 mg/ml; Sigma, St. Louis, MO). After performing electrophoresis, the gel was then soaked in 2% Triton X-100 with gentle shaking for 60 min with single change of detergent solution. The gel was rinsed and next incubated overnight in substrate buffer (50 mM Tris–HCl, pH 7.5, 5 mM $CaCl_2$, and 0.02% NaN_3). Following the incubation, the gel was stained with 0.05%

Coomassie brilliant blue G-250 and then destained in 10% acetic acid and 20% methanol. The gel was photographed and then measured by densitometry.

Western blotting

Melanoma cells were collected and rinsed with PBS, and lysed with RIPA lysis buffer. The lysates were centrifuged at 14,000 rpm for 15 min. The protein concentrations across samples were checked by Bradford method. The samples were denatured by adding SDS running buffer (0.2% bromophenol blue, 4% SDS, 100 mM Tris [pH 6.8], and 20% glycerol) and β-mercaptoethanol. The samples were analyzed by SDS-PAGE on 12% gels. After the proteins were transferred to nitrocellulose membrane, FAK (Abcam, Eugene, OR), pY397 FAK (Abcam, Eugene, OR), Src (Santa Cruz, Dallas, TX), pY416 Src (Cell Signaling Technology, Danvers, MA), RhoA (Abcam, Eugene, OR), pS19 MLC (Cell Signaling Technology, Danvers, MA), MLC (Cell Signaling Technology, Danvers, MA), E-cadherin (Cell Signaling Technology, Danvers, MA), N-cadherin (Santa Cruz, Dallas, TX), and vimentin (Abcam, Eugene, OR) were detected with corresponding primary monoclonal antibodies followed by HRP conjugated secondary antibodies. The labeled proteins were visualized using a chemilumenscence kit.

Rho activation assay

Rho activation assay kit (Upstate Biotechnology, Lake Placid, NY) was employed to determine the activity of RhoA in melanoma cells. Briefly, cell lysates were incubated with rhotekin Rho binding peptide coated on agarose beads for 1 hr, and the GTP-Rho bound to rhotekin-agarose beads were immunoprecipitated and subject to Western blotting detection with anti-RhoA mouse IgG antibody (Santa Cruz).

Immunofluorescence

miRNA antagomir and/or overexpressing plasmid-transfected melanoma cells were grown on cover slips coated with fibronectin (1 μg/ml) before being treated with cordycepin. Cells were then washed with PBS and fixed with 5% paraformaldehyde for 10 min. Cells were permeabilized with 0.3% Triton X-100 in PBS and blocked for 30 min with 5% BSA. Subsequently, cover slips were incubated with anti-E-cadherin, anti-N-cadherin or anti-paxillin for 1 hr at room temperature. This was followed by staining with Alexa 555 or Alexa 488-conjugated anti-rabbit IgG. To image actin filaments, rhodamine-phalloidin (1:40; Life Technologies, Carlsbad, CA) was incubated with cells. Finally, fluorescent staining was visualized with IX71 Olympus inverted microscopy (Olympus, Inc.) with 40X magnification. Colocalization of paxillin and actin was processed by Image J. To analyze the size and number of paxillin-containing focal adhesions, images were background subtracted before thresholding and segmentation were conducted to detect the edges of focal adhesions. Then, the mean size (in pixels) and number of focal adhesions in each cell were calculated.

SUPPLEMENTARY FIGURE

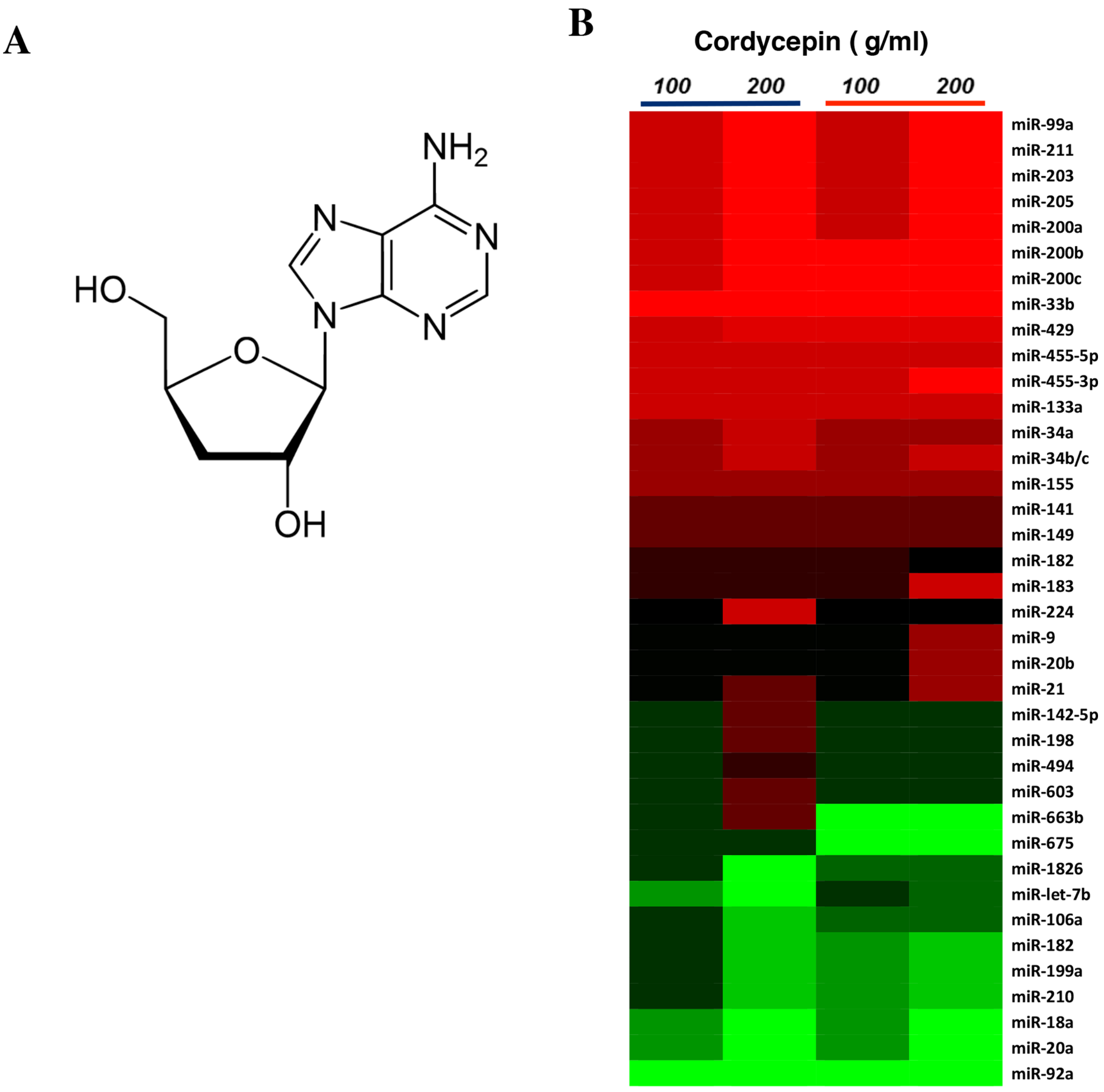

Supplementary Figure 1: Cordycepin promotes changes of miRNA expression profiles in melanoma cells. **(A)** The structure of cordycepin. **(B)** Untreated (control), 100 μg/ml cordycepin-treated, and 200 μg/ml cordycepin-treated A375 (blue line) and Lu1205 (red line) cells were collected, and total cellular RNA was purified. The RNA samples were subjected to miRNAarray analysis. miRNA demonstrating more than 2-fold changes were selected. The heat map images represent the types and expression levels (relative to control) of miRNAs which were either upregulated (red) or downregulated (green).
